# Sustained treatment success at 12 months for drug-resistant TB patients on concomitant bedaquiline-delamanid

**DOI:** 10.5588/ijtldopen.24.0600

**Published:** 2025-02-01

**Authors:** V. Chavan, A. Silsarma, R. Mahajan, S. Khan, P. Singh, A. Iyer, A. Dalal, H. Spencer, P. Isaakidis

**Affiliations:** ^1^ Médecins Sans Frontières, Mumbai, India;; ^2^ Jupiter Hospital, Mumbai, India;; ^3^ Southern African Medical Unit, Médecins Sans Frontières, Cape Town, South Africa.

**Keywords:** tuberculosis, DR-RB, QoL, Mumbai, India, LTFU, recurrence

Dear Editor,

An estimated 10.6 million people developed TB worldwide in 2022, including 410,000 cases of multidrug-resistant or rifampicin-resistant TB (MDR/RR-TB), causing 1.3 million deaths.^1^ Although the treatment outcomes of bedaquiline (BDQ) and delamanid (DLM) containing regimens for drug-resistant TB (DR-TB) are well documented,^2–4^ there is limited data on long-term post-TB treatment outcomes, including relapse, sequelae and quality of life (QoL). The recurrence of TB after successful treatment presents a significant challenge. A meta-analysis revealed that the pooled incidence rate of recurrent TB was 2.3/100 person-years at risk, with rates of 1.5 in low TB burden and 4.1 in high TB burden settings.^5^ In a study by Surie et al. in Chennai, India, 9% of MDR-TB patients who achieved treatment success died, with no cases of recurrent TB observed during long-term follow-up.^6^ In another study in Mumbai, India, the post-treatment case-fatality ratio was 1% at 6 months and 1.7% at 12 months, and the TB recurrence rate was 0.4% at 6 and 0.8% at 12 months.^7^ A multi-country study estimated a 6-month post-treatment TB-recurrence risk of 7.4/1,000 MDR-TB patients, considering deaths as non-recurrences.^8^ Guidelines for the programmatic management of DR-TB in India recommend post-treatment follow-up for up to 24 months for all successfully treated TB patients. However, systematic follow-up of these patients is still lacking.^9^ Recently, the WHO has proposed an optional definition of ‘sustained treatment success’ (alive and TB free) for use in operational research for DR-TB patients assessed at 12-months post-treatment.^10^

A Médecins Sans Frontières (MSF) clinic (Govandi East, Mumbai, India) provides free treatment for DR-TB patients with extensive resistance patterns who need the latest drugs and have limited treatment options. The clinic provides BDQ- and DLM-containing regimens with or without carbapenem to DR-TB patients as part of a ‘salvage’ regimen’ for 18–24 months, based on drug susceptibility testing, adverse events and previous drug exposure.^3,4^ All patients are followed up at 6 months and 12 months to assess sustained treatment success after completion of treatment. During these visits, patients are screened by nurses, examined by a doctor and relevant investigations are advised. Sputum samples from previous pulmonary TB patients are sent for culture. We describe 12-month follow-up outcomes of patients with DR-TB who had failed previous regimens and were successfully treated with concurrent BDQ and DLM plus/minus carbapenem-based regimens. The post-TB follow-up data from patients were entered in an MS Excel (Microsoft, Seattle, WA, USA) spreadsheet and analysed using R v4.4.0 (R Computing, Vienna, Austria). Categorical variables were described using counts and percentages, and continuous variables were described using median and interquartile range (IQR). Ethics approval was obtained from the ethics review board of Jupiter Hospital, Mumbai, India. The study met the criteria for a posteriori analysis of routinely collected clinical data and exempted from MSF Ethical Review Board full review.

From January 2016 to December 2019, 226 patients were treated with regimens containing BDQ and DLM, plus/minus carbapenem. Among these, 141 patients (62%) achieved treatment success: 116 (82%) patients were cured and 25 (18%) completed treatment (Table). Among the successfully treated patients, the median age was 24 years (IQR 20–30); 12 (9%) were less than 15 years old; 88 (62%) were female, two (1.4%) were living with HIV, and one (0.7%) had diabetes mellitus. A total of 108 (77%) and 33 (23%) patients had pre-extensively and extensively drug-resistant TB (XDR-TB), respectively. Overall, 120 (85%) had pulmonary TB, 17 (12%) had extrapulmonary TB and 4 (2.8%) had disseminated TB. Fifty-seven (43%) were severely underweight (BMI <16.5 kg/m^2^). The median treatment duration was 19 months (IQR 18–22): 18 months (IQR 17–20) for BDQ, 19 months (IQR 18–21) for DLM and 7 months (IQR 6–9) for carbapenem. Post-treatment completion at 6 months, 98 patients had sustained treatment success, with 70 evaluated with culture or X-ray and 28 clinically. Of the remaining 43, 38 were lost to follow-up (LTFU), 3 died and 2 had TB recurrences. In the worst-case scenario, assuming all 38 LTFU cases had recurrence or death (classified as treatment failure), the sustained treatment success rate was 69.5% (98/141). In the best-case scenario, assuming all 38 LTFU patients had favourable outcomes, the sustained treatment success rate was 96.5% (136/141). Replicating the calculations for the worst-case scenario at 12 months follow-up, the sustained treatment success was calculated at 53.2% (75/141), and for the best-case scenario, the sustained treatment success was 94.3% (133/141).

**Table.** Six and 12-month post-treatment outcomes of TB patients treated with concomitant BDQ and DLM stratified by baseline clinical and demographic characteristics.

		6-month follow-up				12-month follow-up			
Characteristics	Overall *n* (%)	Sustained treatment success *n* (%)	TB recurrence *n* (%)	Died *n* (%)	Lost to follow-up *n* (%)	Sustained treatment success *n* (%)	TB recurrence *n* (%)	Died, *n* (%)	Lost to follow-up *n* (%)
Overall, *n*	141	98 (70)	2 (1.4)	3 (2.1)	38 (27)	75 (53)	5 (3.5)	3 (2.1)	58 (41)
Age, years, median [IQR]	24 [20–30]	23 [20–29]	24 [22–27]	28 [25–47]	25 [19–30]	23 [20–29]	20 [20–29]	28 [25–47]	24 [20–30]
Females	88 (62.4)	65 (66.3)	0 (0)	3 (100)	20 (52.6)	45 (60.0)	3 (60.0)	3 (100)	37 (63.8)
PLHIV	2 (1.4)	0 (0)	0 (0)	1 (33.3)	1 (2.6)	1 (1.3)	0 (0)	1 (33.3)	0 (0)
Previously treated	121 (85.8)	87 (88.8)	2 (100)	3 (100)	29 (76.3)	67 (89.3)	4 (80.0)	3 (100)	47 (81.0)
Regimen									
BDQ+DLM+IMP/MPM	87 (61.7)	61 (62.2)	2 (100)	2 (67.7)	22 (57.9)	45 (60.0)	4 (80.0)	2 (66.7)	36 (62.1)
BDQ+DLM	51 (36.2)	35 (35.7)	0 (0)	1 (33.3)	15 (39.5)	28 (37.3)	1 (20.0)	1 (33.3)	21 (36.2)
BDQ+DLM+MPM	3 (2.1)	2 (2.0)	0 (0)	0 (0)	1 (2.6)	2 (2.7)	0 (0)	0 (0)	1 (1.7)
BDQ-exposed	10 (7.1)	8 (8.2)	0 (0)	1 (33.3)	1 (2.6)	7 (9.3)	0 (0)	1 (33.3)	2 (3.4)
Resistance profile									
Pre-XDR-TB	108 (76.6)	77 (78.6)	1 (50)	2 (67.7)	28 (73.7)	58 (77.3)	3 (60.0)	2 (66.7)	45 (77.6)
XDR-TB	33 (23.4)	21 (21.4)	1 (50)	1 (33.3)	10 (26.3)	17 (22.7)	2 (40.0)	1 (33.3)	13 (22.4)
Site of infection									
Pulmonary	120 (85.1)	83 (84.7)	2 (100)	2 (67.7)	33 (86.8)	61 (81.3)	5 (100)	2 (66.7)	52 (89.7)
Extrapulmonary	17 (12.1)	12 (12.2)	0 (0)	1 (33.3)	4 (10.5)	11 (114.7)	0 (0)	1 (33.3)	5 (8.6)
Disseminated	4 (2.8)	3 (3.1)	0 (0)	0 (0)	1 (2.6)	3 (4.0)	0 (0)	0 (0)	1 (1.7)
Nutritional status									
Severely underweight	57 (42.9)	32 (33.7)	2 (100)	2 (67.7)	21 (63.6)	25 (34.2)	4 (100)	2 (66.7)	26 (49.1)
Missing data, *n*	8	3	0	0	5	2	1	0	5
BMI, kg/m^2^, median [IQR]	18.1 [15.0–20.3]	18.2 [15.9–20.3]	NA	15.2 [13.7–16.7]	16.1 [13.8–19.6]	18.1 [15.9–20.2]	12.1 [12.1–12.1]	15.2 [13.7–16.7]	18.1 [14.5–21.6]
Missing data, *n*	24	8	2	1	13	5	4	1	14

BDQ = bedaquiline; DLM = delamanid; IQR = interquartile range; PLHIV = people living with HIV; IMP = imipenem; MPM = meropenem; XDR = extensively drug-resistant tuberculosis; BMI = body mass index; NA = not available.

Our study demonstrates that in the best-case scenario, those receiving concomitant BDQ and DLM, with or without carbapenem, showed a promising sustained treatment success of 94% at 12 months post-treatment. However, 41% of patients in our cohort were LTFU by 12 months post-treatment. Patients reported having moved out of the area, lack of money and lack of symptoms as reasons for not attending 6- and 12-month follow-up appointments. Some studies employ inverse probability weighting to account for LTFU.^7,8^ In a study to estimate post-treatment recurrence after MDR-TB treatment, the exclusion of patients with missing follow-up data without inverse-probability weighting had minimal impact on estimates.^8^ We believe that the LTFU in our study likely had a limited impact on the sustained treatment success rates, but we also present a worst-case scenario (assuming all LTFU cases as unfavourable outcomes), which significantly underestimates the sustained treatment success rates. Therefore, these results should be interpreted with caution. Nevertheless, we believe the true sustained treatment success rates are closer to the best-case scenario, with 96.5% at 6 months and 94.3% at 12 months. Our sustained treatment success rates are slightly lower than those estimated by Huddart et al. in Mumbai.^7^ This difference could be due to our focus on highly selected, high-risk DR-TB patients treated with newer regimens. All three deaths in our study occurred within the first 6 months, but we were unable to determine the cause of death. Additionally, 2 out of 5 TB recurrences happened within the first 6 months post-treatment. Notably, 4 of the 5 patients with TB recurrence were severely underweight (BMI <16.5 kg/m^2^), with the BMI of one patient unknown. Moreover, 2 out of the 3 deaths were also severely underweight. Although the small sample size limits our ability to analyse the impact of undernutrition on post-treatment sustained treatment success rates, our data suggest a likely link between poor nutrition and these outcomes. Further larger studies in this area are needed and should also include cost analyses, patient perspectives, and disability and QoL indicators.

In conclusion, we recommend that programmatic efforts for post-treatment follow-ups be enhanced, requiring sustained commitment and funding for TB programmes.
